# Clinical Trial Enrollment, Ineligibility, and Reasons for Decline in Older vs Younger Patients With Cancer in the National Cancer Institute Community Oncology Research Program

**DOI:** 10.1001/jamanetworkopen.2022.35714

**Published:** 2022-10-10

**Authors:** Mina S. Sedrak, Jingran Ji, Abhay Tiwari, Supriya G. Mohile, William Dale, Jennifer G. Le-Rademacher

**Affiliations:** 1 Department of Medical Oncology and Therapeutics Research, City of Hope Comprehensive Cancer Center, Duarte, California; 2 Department of Medicine, University of Rochester, Rochester, New York; 3 Department of Quantitative Health Sciences, Mayo Clinic, Rochester, Minnesota

## Abstract

This cross-sectional study assesses differences in clinical trial participation among older vs younger adults with cancer.

## Introduction

Although barriers to older adult participation in cancer clinical trials are frequently studied, enrollment has not changed over time.^1^ One reason for this lack of change is that few studies have examined barriers that hinder older adult enrollment in community settings, where more than 80% of older adults receive their cancer care.^2^ We sought to compare enrollment patterns, ineligibility, and reasons for decline among older and younger adult cancer patients in community settings.

## Methods

We examined a clinical trial screening logs database from January 1, 2016, to December 31, 2019, in the National Cancer Institute (NCI) Community Oncology Research Program (NCORP).^3^ The NCI developed screening logs to collect data obtained by NCORP research staff during active screening. Logs included trial characteristics, enrollment (yes or no), and reasons for nonenrollment (ineligible vs eligible but the patient declined). Additional reasons why participants were ineligible or eligible but chose not to enroll were also identified. This analysis was determined by City of Hope Institutional Review Board to be exempt from review because it included only deidentified administrative data. This cross-sectional study followed the STROBE reporting guideline.^4^

We included and grouped entries in 2 age cohorts: younger (age 50-69 years) and older (age ≥70 years). We chose the cut-off of 70 years because those older than 70 years are severely underrepresented in both NCI and US Food and Drug Administration (FDA) trials.^5^ We chose to include ethnicity as a study variable because White patients continue to be overrepresented in cancer clinical trials, whereas racial and ethnic minority subgroups are underrepresented. Patient characteristics, enrollment rates, and reasons for nonenrollment were compared by age group using χ^2^ tests. A 2-sided *P* < .05 was considered to be statistically significant.

## Results

Demographic, clinical, and trial characteristics are given in the Table. Of 2298 patients screened and offered a clinical trial, 1709 (74%) were younger and 589 (26%) were older adults (*P* < .001). Among all approached patients, 1540 (67%) enrolled, and enrollment rate did not differ by age group (68% [1156/1709] younger vs 65% [384/589] older; *P* = .28). Reasons for nonenrollment included being ineligible (18% [315/1709] younger vs 23% [133/589] older) or being eligible but declined (13% [224/1709] younger vs 11% [67/589] older). The most common reasons for ineligibility among both groups were the presence of comorbidity (23% [72/315] younger vs 26% [35/133] older), failure to meet the protocol-specific stage and cancer histologic stage (30% [95/315] younger vs 24% [32/133] older), and biomarker criteria not met (7% [22/315] younger vs 13% [17/133] older) (*P* = .004) (Figure, A). Among both groups, the most common reasons for eligible patients to decline enrollment were perceived adverse effects being too great (22% [49/224] younger vs 21% [14/67] older) and no desire to participate in research (20% [45/224] younger vs 18% [12/67] older) (*P* = .85) (Figure, B).

**Table.** Participant Demographic, Clinical, and Trial Characteristics by Age Group^a^

Characteristic	Age category, y	
	50-69 (n = 1709)	≥70 (n = 589)
Age, mean (SD) [range], y	60.0 (5.7) [50-69]	74.7 (4.4) [70-92]
Sex		
Female	1371 (80)	375 (64)
Male	338 (20)	214 (36)
Ethnicity		
Hispanic or Latinx	96 (6)	16 (3)
Not Hispanic or Latinx	1605 (94)	563 (96)
Unknown	8 (0.5)	10 (2)
Marital status		
Married or domestic partnership	1142 (67)	378 (64)
Divorced or separated	335 (20)	80 (14)
Widowed	98 (6)	111 (19)
Never married	134 (8)	20 (3)
Rural site	364 (21)	143 (24)
Educational level^b^		
Less than high school graduate	99 (6)	53 (9)
High school graduate	396 (23)	186 (32)
College or greater	1203 (70)	344 (58)
Employment		
Working	827 (49)	56 (10)
Not working, not retired	387 (22)	495 (84)
Retired	486 (29)	38 (6)
Income, $		
≤50 000	575 (34)	279 (47)
≥51 000	773 (45)	174 (30)
Patient did not respond	361 (21)	136 (23)
No. of comorbidities		
0	650 (38)	99 (17)
1	539 (32)	188 (32)
≥2	520 (30)	302 (51)
Type of comorbidities		
Hypertension	721 (42)	365 (62)
Hypercholesterolemia	446 (26)	224 (38)
Heart disease	133 (8)	120 (20)
Diabetic neuropathy	38 (2)	23 (4)
Other cancer	73 (4)	46 (8)
Other systemic	184 (11)	77 (13)
Cancer type^b^		
Breast	1073 (63)	178 (30)
Lung	358 (21)	252 (43)
Genitourinary	15 (0.9)	49 (8)
Gastrointestinal	8 (0.5)	38 (6)
Hematologic	10 (0.6)	12 (2)
Other	125 (7.6)	57 (9)
Cancer stage^b^		
I-III	1484 (96)	464 (92)
IV	62 (4)	38 (8)
Clinical trial type^b^		
Therapeutic clinical trial	798 (53)	308 (58)
Nontherapeutic clinical trial	723 (48)	220 (42)

^a^ Data are presented as number (percentage) of study participants unless otherwise indicated.

^b^ Some row percentages may not total 100% because of rounding and missing data, including 111 missing cancer types, 87 missing disease stages, 61 missing clinical trial types, 6 missing educational levels, and 3 missing diagnoses.

**Figure.**
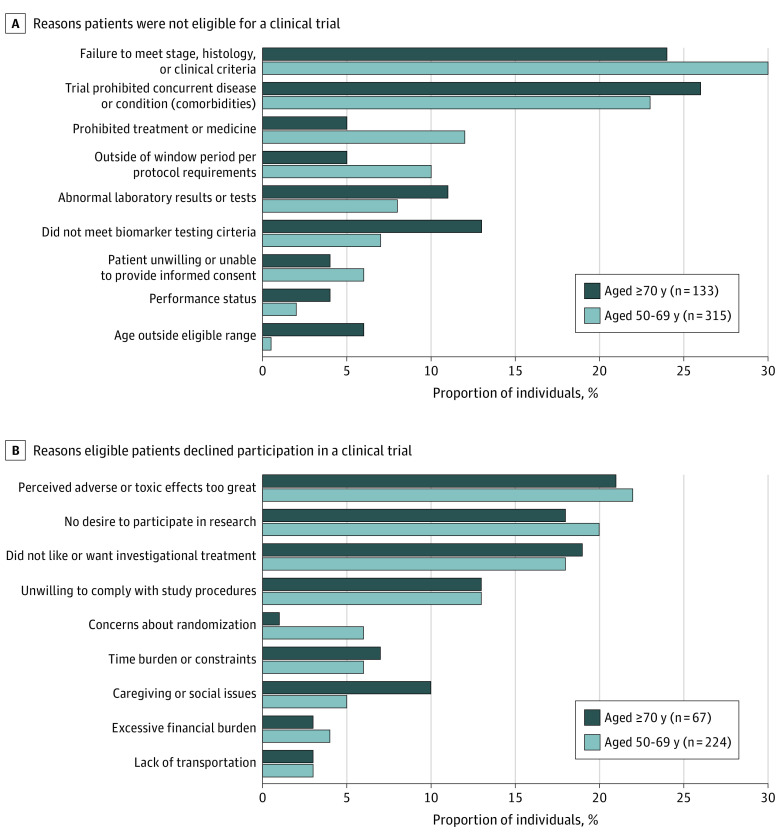
Reasons Patients Did Not Enroll in a Clinical Trial A, Reasons patients were not eligible for a clinical trial. B, Reasons eligible patients declined participation in a clinical trial. Although the reasons for nonenrollment were similar across age groups when eligible patients declined enrollment (*P* = .85), the reasons for ineligibility differed by age group (*P* = .004).

## Discussion

Despite differences in education, income, and comorbid conditions, when screened and offered a clinical trial, enrollment rates among adult cancer patients in community settings were similar independent of age. Older adults were as willing to participate as their younger counterparts. Among eligible patients, the reasons for declining enrollment were similar across age groups; however, reasons for ineligibility differed. This study included actively screened cancer patients with access to clinics that offer NCI clinical trials, which limits generalizability. Nonetheless, our findings underscore that the disparity in cancer research participation among older adults is driven by factors upstream of the patient, such as restrictive eligibility criteria.

Efforts are under way to broaden eligibility criteria to make trials more generalizable for patients of all ages, including initiatives by the American Society of Clinical Oncology, Friends of Cancer Research, and the FDA.^6^ How these initiatives will help the geriatric population is not yet known. Given the rapidly aging population, this is a crucial time to address the multiple barriers to clinical trial participation in the geriatric population to ensure all patients with cancer receive the highest-quality, evidence-based care.
